# Molecular Cytogenetics in Trough Shells (Mactridae, Bivalvia): Divergent GC-Rich Heterochromatin Content

**DOI:** 10.3390/genes7080047

**Published:** 2016-08-16

**Authors:** Daniel García-Souto, Concepción Pérez-García, Jack Kendall, Juan J. Pasantes

**Affiliations:** Departamento de Bioquímica, Xenética e Inmunoloxía, Universidade de Vigo, E-36310 Vigo, Spain; danielgarciasouto@gmail.com (D.G.-S.); concepcionperezgar@gmail.com (C.P.-G.); jk.kendall@hotmail.com (J.K.)

**Keywords:** trough shells, chromosome, heterochromatin, fluorescent in situ hybridization, histone genes, ribosomal RNA genes

## Abstract

The family Mactridae is composed of a diverse group of marine organisms, commonly known as trough shells or surf clams, which illustrate a global distribution. Although this family includes some of the most fished and cultured bivalve species, their chromosomes are poorly studied. In this work, we analyzed the chromosomes of *Spisula solida*, *Spisula subtruncata* and *Mactra stultorum* by means of fluorochrome staining, C-banding and fluorescent in situ hybridization using 28S ribosomal DNA (rDNA), 5S rDNA, H3 histone gene and telomeric probes. All three trough shells presented 2n = 38 chromosomes but different karyotype compositions. As happens in most bivalves, GC-rich regions were limited to the nucleolus organizing regions in *Spisula solida*. In contrast, many GC-rich heterochromatic bands were detected in both *Spisula subtruncata* and *Mactra stultorum*. Although the three trough shells presented single 5S rDNA and H3 histone gene clusters, their chromosomal locations differed. Regarding major rDNA clusters, while *Spisula subtruncata* presented a single cluster, both *Spisula solida* and *Mactra stultorum* showed two. No evidence of intercalary telomeric signals was detected in these species. The molecular cytogenetic characterization of these taxa will contribute to understanding the role played by chromosome changes in the evolution of trough shells.

## 1. Introduction

The family Mactridae (Lamarck 1809) is composed of a diverse group of marine organisms, commonly known as trough shells, duck clams or surf clams, showing a global distribution [1] and including some of the most fished and cultured bivalve species. Although trough shells are among the better known bivalves, some questions about their biology are not clear. In contrast with most other bivalve families in which many genera are available, in the family Mactridae there are only a few recognized genera and some groups do not match to any of them. Phylogenetic relationships among species of this family, and among specimens of single putative species, were the subject of some recent investigations using DNA sequences [2,3,4,5], giving increasing evidence of cryptic speciation in the group [2,4].

The chromosomal characterization of trough shells is far beyond the knowledge achieved for other bivalve families [6,7]. Classical studies were limited to determining chromosome numbers in a few species [8,9,10,11,12]. More recently, the karyotypes of the dwarf surf clam *Mulinia lateralis* [13], the fat gaper (horse clam) *Tresus capax* (*Lutraria maxima*) [14,15], and the sunray surf clam *Mactra chinensis* [16] were described. Regarding molecular cytogenetic techniques, fluorescent in situ hybridization (FISH) was only applied to map telomeric sequences [17] and major ribosomal DNAs (rDNAs) [18] to the chromosomes of *Mulinia lateralis*.

In order to cytogenetically characterize the trough shells *Spisula solida*, *Spisula subtruncata* and *Mactra stultorum*, we studied their chromosomes by means of chromomycin A3 (CMA)/4′,6-diamidino-2-phenylindole (DAPI) and DAPI/propidium iodide (PI) fluorescence staining, C-banding and FISH using major rDNA, 5S rDNA, H3 histone genes and telomeric probes. Our results highlighted chromosomal similarities and differences among these taxa that can help to understand the role played by chromosome changes in the evolution of trough shells.

## 2. Experimental Section 

### 2.1. Trough Shell Specimens

Samples of the thick trough shell *Spisula solida* (Linnaeus 1758), the cut trough shell *Spisula subtruncata* (da Costa 1778) and the rayed trough shell *Mactra stultorum* (Linnaeus 1758) were collected from natural populations in Ría de Pontevedra and Ría de Vigo (NW Spain).

### 2.2. Chromosome Preparation, Fluorochrome Staining and C-Banding

Chromosome preparations were obtained from gill and gonadic tissues following previously published methods [19,20]. After exposing the trough shells overnight to colchicine (0.005%), gills and gonads were removed, treated with 50% (20 min) and 25% (20 min) sea water and fixed in ethanol/acetic acid (3:1) three times for 20 min each time. Small pieces of the fixed tissues were dissociated in 60% acetic acid and the cell suspensions were dropped onto slides heated to 50 °C. 

Fluorochrome staining was performed as previously described [21,22]. Some of the chromosome preparations were stained with CMA (0.25 mg/mL, 2 h), counterstained with DAPI (0.14 μg/mL, 8 min) and mounted with antifade (Vectashield, Vector, Burlingame, USA). After visualization and photography, the same chromosome preparations were re-stained with a combination of DAPI and PI (0.07 μg/mL, 8 min), mounted in antifade and photographed again. Chromosome counts were performed in at least 200 metaphase plates obtained from 10 specimens (5 males, 5 females) of each species. To detect heterochromatic regions on these species, some preparations were also C-banded using barium hydroxide [23] and stained with acridine orange (0.1 mg/mL).

### 2.3. DNA Extraction, PCR Amplification and Probe Labeling

Total genomic DNA was extracted from ethanol-preserved adductor muscles following a classical method [24] with slight modifications [25]. Small pieces of tissue were homogenized in hexadecyltrimethylammonium bromide (CTAB) buffer and digested with pronase (1.5 mg/mL, 60 °C, overnight) (Sigma Aldrich, St. Louis, MO, USA) and RNase A from bovine pancreas (1 mg/mL, 37 °C, 1 h) (Sigma Aldrich, St. Louis, MO, USA). The DNA was extracted with phenol:chloroform:isoamyl alcohol (24:24:1) and stored at 4 °C until further use. 

FISH probes were generated by polymerase chain reaction (PCR) in 20 μL reactions containing 50 ng DNA, 1× PCR buffer, 0.5 mM of each dNTP (Thermo Fisher Scientific. Waltham, MA, USA), 2.5 mM MgCl_2_, 1 μM of each primer and 1 U BIOTAQ DNA polymerase (Bioline, London, UK). As shown in Table 1, universal primers [26] were used to amplify a fragment of the 28S rRNA gene of the major rDNA repeat. The whole repeat of the 5S rDNA was amplified using primers described in [27]. H3 histone genes were amplified using the primers proposed by [28]. Following a denaturation step at 95 °C for 5 min, 30 cycles of amplification (Table 1) and a final extension of 7 min at 72 °C were performed in a GeneAmp PCR system 9700 (Applied Biosystems. Foster City, CA, USA). PCR products were checked by electrophoresis in 2% agarose gels. 28S rDNA probes were labeled with biotin-16-dUTP (Roche Applied Science, Penzberg, Germany) and/or digoxigenin-11-dUTP (10× DIG Labeling Mix, Roche Applied Science) by nick translation (Roche Applied Science. Penzberg, Germany). H3 histone gene and 5S rDNA probes were labeled with either biotin-16-dUTP (20 μM) or digoxigenin-11-dUTP (5 μM) by PCR. The labeled PCR products were precipitated before FISH.

### 2.4. Fluorescent in Situ Hybridization (FISH)

Single- and double-color FISH and re-hybridization experiments were performed as previously published [29]. Preparations were digested with RNase A (Sigma Aldrich, St. Louis, MO, USA) and pepsin (Sigma Aldrich, St. Louis, MO, USA) before denaturation (70 °C, 2 min). After overnight hybridization at 37 °C, biotin-labeled probes were detected with fluorescein avidin and biotinylated mouse anti-avidin antibodies (Vector. Burlingame, CA, USA) and digoxigenin-labeled ones were detected with mouse anti-digoxigenin and anti-mouse rhodamine antibodies (Sigma Aldrich, St. Louis, MO, USA). Slides were counterstained with DAPI (0.14 μg/mL in 2× saline sodium citrate) and mounted in antifade (Vectashield, Vector, Burlingame, CA, USA). FISH with the vertebrate telomeric (C_3_TA_2_)_3_ peptide nucleic acid (PNA) probe (Applied Biosystems) was performed following the protocol indicated by the supplier.

A minimum of 20 metaphase plates per probe or combination of probes in 10 specimens (5 male, 5 female) per species were recorded using a Nikon Eclipse-800 microscope (Tokio, Japan) equipped with an epifluorescence system. Separated images for each fluorochrome were obtained with a DS-Qi1Mc CCD camera (Nikon) controlled by the NIS-Elements software (Nikon). Merging of the images was performed with Adobe Photoshop CS2 (San Jose, CA, USA). 

Ten of the best metaphase plates showing FISH signals were used to construct karyotypes for each species. Chromosome and arm lengths were carefully measured and relative lengths and centromeric indices were determined. 

## 3. Results

**Figure 1:**
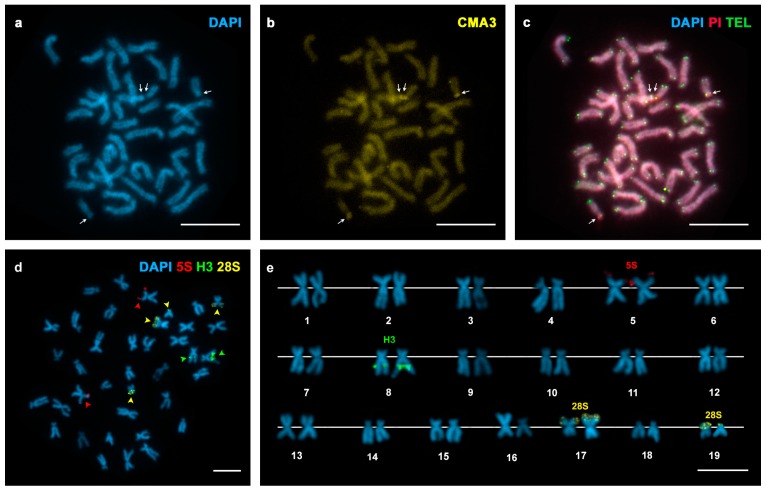
Fluorochrome staining and fluorescent in situ hybridization (FISH) mapping of telomeric (TEL), ribosomal DNA (rDNA) and histone gene probes to mitotic chromosomes of *Spisula solida*. (**a**) 4′,6-diamidino-2-phenylindole (DAPI)-stained metaphase plate showing DAPI dull regions on the short arms of two chromosome pairs (arrows); (**b**) Chromomycin A3 (CMA) staining of the same metaphase plate showed that DAPI dull regions were CMA bright (arrows); (**c**) FISH mapping of telomeric sequences to the same metaphase plate counterstained with DAPI/propidium iodide (PI) shows signals on the telomeres. Note that the four DAPI negative regions are stained with PI (arrows); (**d**) FISH mapping of 28S rDNA (yellow arrowheads), 5S rDNA (red arrowheads) and H3 histone gene (green arrowheads) probes on a metaphase counterstained with DAPI and the corresponding karyotype (**e**). Scale bars = 5 μm.

**Figure 2:**
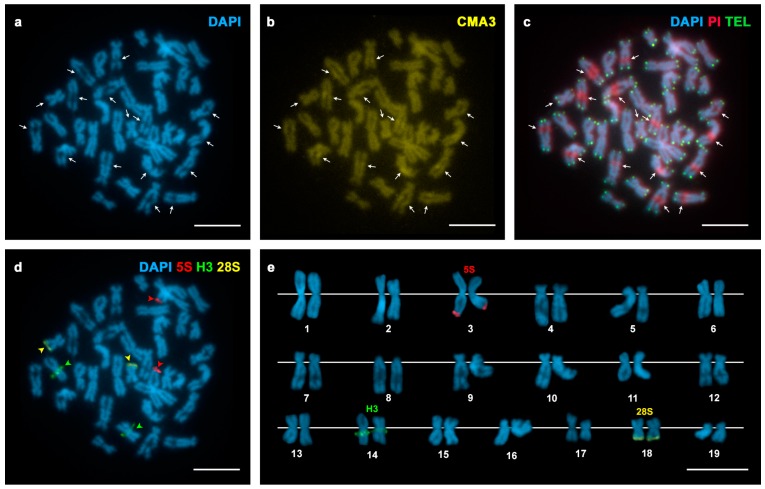
Fluorochrome staining and FISH mapping of telomeric, rDNA and histone gene probes to mitotic chromosomes of *Spisula subtruncata*. (**a**) DAPI-stained metaphase plate showing DAPI dull regions (arrows) subterminal (1) and intercalary (7) to the long arms of eight chromosome pairs; (**b**) CMA staining of the same metaphase plate showed that the DAPI dull regions were stained with CMA (arrows); (**c**) FISH mapping of telomeric sequences to the same metaphase plate counterstained with DAPI/PI shows green signals at the ends of all chromatids and DAPI−/PI+ regions stained in red (arrows); (**d**) FISH mapping of 28S rDNA (yellow arrowheads), 5S rDNA (red arrowheads) and H3 histone gene (green arrowheads) probes on the same metaphase counterstained with DAPI and the corresponding karyotype (**e**). Scale bars = 5 μm.

**Figure 3:**
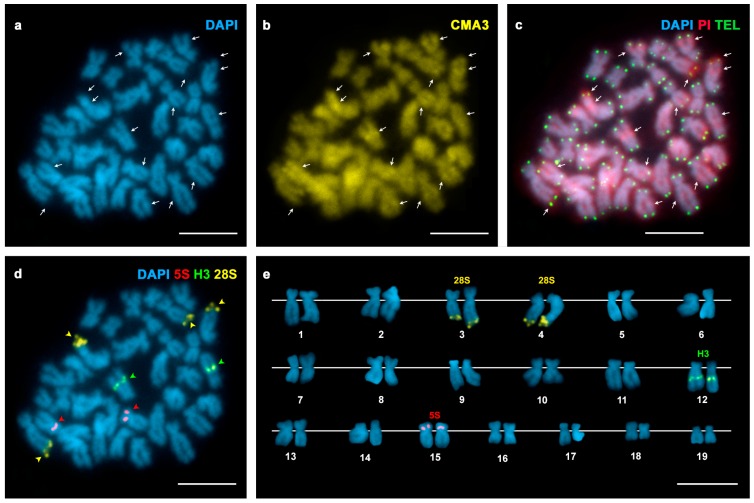
Fluorochrome staining and FISH mapping of telomeric, rDNA and histone gene probes to mitotic chromosomes of *Mactra stultorum*. (**a**) DAPI-stained metaphase plate showing DAPI dull regions (arrows) subterminal (2) and intercalary (6) to the long arms of seven chromosome pairs; (**b**) CMA staining of the same metaphase plate showed that the DAPI dull regions were stained with CMA (arrows); (**c**) FISH mapping of telomeric sequences to the same metaphase plate counterstained with DAPI/PI shows green signals at the ends of all chromatids and DAPI−/PI+ red-stained regions (arrows); (**d**) FISH mapping of 28S rDNA (yellow arrowheads), 5S rDNA (red arrowheads) and H3 histone gene (green arrowheads) probes on the same metaphase plate counterstained with DAPI and the corresponding karyotype (**e**). Scale bars = 5 μm.

The diploid chromosome numbers determined for *Spisula solida*, *Spisula subtruncata* and *Mactra stultorum* were 2n = 38 (Figure 1, Figure 2 and Figure 3). *Spisula solida* showed a karyotype composed of five metacentric, four meta/submetacentric, three submetacentric, three submeta/subtelocentric and four subtelocentric chromosome pairs (Figure 1e). The karyotype of *Spisula subtruncata* presented eight metacentric, one meta/submetacentric, two submetacentric, three submeta/subtelocentric, four subtelocentric and one telocentric chromosome pairs (Figure 2e). In *Mactra stultorum*, the karyotype was composed of five metacentric, three meta/submetacentric, four submetacentric, four submeta/subtelocentric, and three subtelocentric chromosome pairs (Figure 3e).

The presence of AT- and/or GC-rich chromosomal regions was examined using a combination of AT-specific (DAPI), GC-specific (CMA) or unspecific (PI) fluorochromes. DAPI staining revealed two DAPI negative regions, subterminal to the short arms of two chromosome pairs in *Spisula solida* (Figure 1a); these regions were clearly stained with both CMA and PI (Figure 1b,c). In contrast, *Spisula subtruncata* showed eight chromosome pairs displaying DAPI−/CMA+ regions, seven of them at intercalary locations and one subterminal (Figure 2a–c). *Mactra stultorum* showed six pairs of intercalary and two pairs of subterminal DAPI−/CMA+ bands (Figure 3a–c). As detected by C-banding (Figure S1), these DAPI−/CMA+ regions were heterochromatic.

FISH experiments using 28S rDNA probes demonstrated that major rDNA clusters were located at subterminal DAPI−/CMA+ regions in these taxa. Both *Spisula solida* and *Mactra stultorum* showed two major rDNA signals, subterminally located on the short arms of chromosome pairs 17 and 19 (Figure 1d,e) and on the long arms of chromosome pairs 3 and 4 (Figure 3d,e), respectively. In contrast, a single major rDNA cluster was detected on the long arms of chromosome pair 18 in *Spisula subtruncata* (Figure 2d,e).

In order to investigate the location of the major rDNA clusters in relation to the 5S rDNA and the core histone gene clusters, we performed double and sequential FISH experiments using 28S rDNA, 5S rDNA and H3 core histone gene probes. All three trough shells showed a single 5S rDNA cluster subterminal on the short arms of chromosome pair 5 in *Spisula solida* (Figure 1d,e) and on the long arms of chromosome pair 3 in *Spisula subtruncata* (Figure 2d,e), and intercalary on the short arms of chromosome pair 15 in *Mactra stultorum* (Figure 3d,e). Although H3 histone gene clusters also mapped to a single locus in the three analyzed species, their locations differed and were intercalary to the long arms of subtelocentric chromosome pairs 8 in *Spisula solida* (Figure 1d,e) and 12 in *Mactra stultorum* (Figure 3d,e) but subcentromeric to the long arms of metacentric chromosome pair 14 in *Spisula subtruncata* (Figure 2d,e). Apart from the histone gene cluster in *Mactra stultorum* chromosome pair 12, neither histone gene nor 5S rDNA clusters were located on the chromosome pairs that bear DAPI−/CMA+ heterochromatic bands.

In order to detect telomeric sequences in these species, we also performed FISH experiments using a vertebrate telomeric (C_3_TA_2_)_3_ PNA as probe. As shown in Figure 1c, Figure 2c and Figure 3c, telomeric signals were detected exclusively at the ends of the sister chromatids of every mitotic chromosome without any indication of intercalary signals.

A summary of the results obtained in this study, together with previously published cytogenetic data in species of the family Mactridae, can be observed in Table 2.

**Figure 4:**
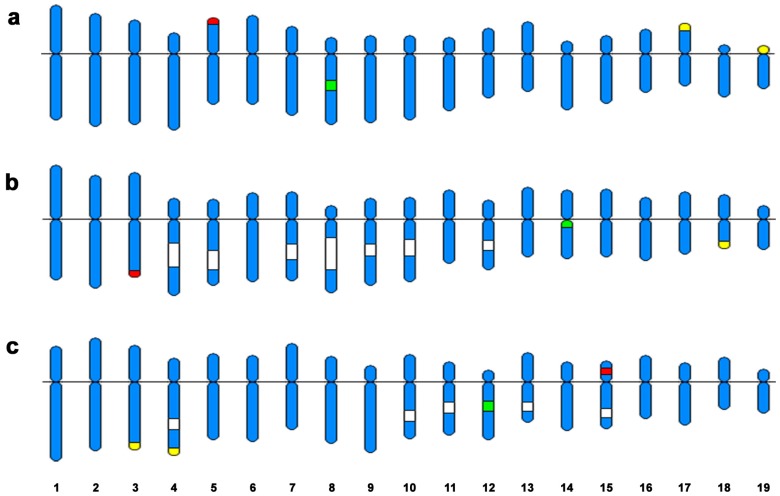
Ideogrammatic karyotypes of the trough shells: (**a**) *Spisula solida*; (**b**) *Spisula subtruncata*; and (**c**) *Mactra stultorum*. DAPI dull (CMA bright/C positive) bands are depicted in white. 28S rDNA signals, drawn in yellow, are coincident with the subterminal DAPI−/CMA+/C+ bands. 5S rDNA clusters are portrayed in red. H3 histone gene clusters, green, are coincident with DAPI−/CMA+/C+ bands in *Mactra stultorum*.

Ideogrammatic representations of the trough shell karyotypes showing the location of the GC-rich heterochromatic regions together with 28S rDNA, 5S rDNA and H3 histone gene clusters are displayed in Figure 4.

## 4. Discussion

Chromosome numbers have been described in only a few species of the family Mactridae (Table 2) [8,9,10,11,12,13,14,15,16,17,18]. The diploid chromosome numbers of 2n = 38 recorded in this work for *Mactra stultorum*, *Spisula solida* and *Spisula subtruncata* are in accordance with previous studies on seven mactrid taxa; however, they do differ from the 2n = 36 reported for *Raeta* (*Labiosa*) *plicatella* [8] and the 2n = 34 described in *Tressus capax* (*Lutraria maxima*) [14,15].

Regarding karyotype composition, striking differences were detected among karyotypes of the species belonging to the family Mactridae. While all chromosome pairs were telocentric in *Mulinia lateralis* [13,17,18], the other three species in which karyotypes were previously described, *Mactra chinensis* [11,16], *Spisula solidissima* [12] and *Tresus capax* (*Lutraria maxima*) [14,15], mostly showed metacentric and submetacentric chromosome pairs. The karyotypes of *Mactra stultorum*, *Spisula solida* and *Spisula subtruncata* here described also presented a high proportion of metacentric and submetacentric chromosome pairs.

The application of diverse combinations of base-specific fluorochromes to the staining of bivalve chromosomes has mostly detected GC-rich regions coincident with the nucleolus organizing regions (NORs) [7,21,22,27,28,29,30,31]. Our results showed that this was also the case for *Spisula solida* in which the two GC-rich regions detected were coincident with the two major rRNA gene clusters. In contrast, the presence of seven intercalary GC-rich regions, in addition to the single GC-rich NOR, in *Spisula subtruncata* and the six intercalary GC-rich regions located outside the two NORs in *Mactra stultorum* are a rare phenomenon in bivalves. In fact, these kind of GC-rich bands were previously reported only for the wedge shell *Donax trunculus* [32,33] and the zebra mussel *Dreissena polymorpha* [34,35]. As also happened in *Donax trunculus* [33], C-banding demonstrated that these GC-rich regions were heterochromatic in both *Spisula subtruncata* and *Mactra stultorum* thus pointing to a probable abundance of GC-rich satellite DNA in these species that is going to be analyzed in the near future. In the meantime, the occurrence of high amounts of intercalary GC-rich heterochromatin in *Spisula subtruncata* and *Mactra stultorum*, but not in *Spisula solida*, suggests that heterochromatin amplification mechanisms (unequal exchange, transposition...) are operating in some trough shell taxa. It is tempting to think that this situation also happens in cryptic species [2,4]; if this were the case, cytogenetic research could contribute to both solving identification issues and establishing phylogenetic relationships within these taxa.

Although multigene families are useful cytogenetic markers for studying chromosomal evolution, their use in bivalves is quite scarce. With respect to the order Veneroida, major rDNA clusters have been previously mapped in 23 species [7,25,33,36,37], 5S rDNA clusters in 14 [7,25], core histone gene clusters in 12 [7] and telomeric sequences in 18 [7,25,33,36,37,38], most of them in species of the family Veneridae. The only species of Mactridae in which the location of some of these sequences was known was *Mulinia lateralis* [17,18]. 

The presence of two 28S rDNA signals subterminal to two chromosome pairs in *Mulinia lateralis* [18] coincided with our results for *Mactra stultorum* and *Spisula solida* but differed with the single pair bearing these signals in *Spisula subtruncata*. With regards to the chromosomal location of 5S rDNA and histone gene clusters, no previous data were available for any species of Mactridae. The occurrence of single minor rDNA and histone gene clusters in the trough shells *Spisula solida*, *Spisula subtruncata* and *Mactra stultorum* (Table 2) is similar to the situation found in some species of venerid clams [7].

Concerning telomeric sequences, the hybridization signals obtained at chromosome ends in *Spisula solida*, *Spisula subtruncata* and *Mactra stultorum* after using the vertebrate telomeric repeat as probe is coincident with results found in most other bivalves, including the mactrid *Mulinia lateralis* [17], and further support the molecular data obtained in *Donax trunculus* showing that bivalve telomeres were constituted by tandem repeats of the hexanucleotide that also constitutes the vertebrate telomeric sequence [38].

In summary, the results obtained in this work highlighted chromosomal similarities and differences among these taxa that can contribute to a better understanding of the role played by chromosome changes in the evolution of trough shells.

## Figures and Tables

**Table 1:** Primers and parameters used in the PCR.

Probe	Sequences of the Primers	Denaturation	Annealing	Elongation
28S rDNA	LR10R: 5′GACCCTGTTGAGCTTGA3′ LR12: 5′GACTTAGAGGCGTTCAG3′	95 °C, 20 s	48 °C, 20 s	72 °C, 30 s
5S rDNA	F: 5′CAACGTGATATGGTCGTAGAC3′ R: 5′AACACCGGTTCTCGTCCGATC3′	95 °C, 20 s	44 °C, 20 s	72 °C, 20 s
H3 histone gene	F: 5′ATGGCTCGTACCAAGCAGACVGC3′ R: 5′ATATCCTTRGGCATRATRGTGAC3′	95 °C, 15 s	48 °C, 15 s	72 °C, 15 s

**Table 2:** Chromosome numbers, karyotypes and fluorescent in situ hybridization (FISH) mapping data in trough shells.

Species	2n	Karyotype	28S rDNA	5S rDNA	Histone Genes	Telomeric Sequences	References
*Mactra chinensis*	38	10 sm, 9 st					[11]
	38	10 m, 8 sm, 1 st/t					[16]
*Mactra stultorum*	38						[10]
	38	5 m, 3 m/sm, 4 sm, 4 sm/st, 3 st	3q ter (st), 4q ter (st)	15p cen (sm)	12q ic (st)	terminal	This study
*Mactra sp.*	36						[8]
*Mulinia lateralis*	36						[8]
	38	19 t					[13]
	38	19 t				terminal	[17]
	38	19 t	15q ter (t), 19q ter (t)				[18]
*Raeta (Labiosa) plicatella*	36						[8]
*Spisula solida*	38	5 m, 4 m/sm, 3 sm, 3 sm/st, 4 st	17p ter (sm), 19p ter (st)	5p ter (sm)	8q ic (st)	terminal	This study
*Spisula solidissima*	38	4 m, 5 sm, 10 st/t					[12]
*Spisula subtruncata*	38	8 m, 1 m/sm, 2 sm, 3 sm/st, 4 st, 1 t	18q ter (m)	3q ter (m)	14q cen (m)	terminal	This study
*Tresus capax*	34	10 m, 7 sm	no signals				[14]
(*Lutraria maxima*)	34	10 m, 6 sm, 1 st/t					[15]

m: metacentric; sm: submetacentric; st: subtelocentric; t telocentric; p: short arm; q: long arm; cen: subcentromeric; ic: intercalar; ter: subterminal.

## Supplementary Materials

The following are available online at www.mdpi.com/2073-4425/7/8/47/s1, Figure S1: C-banding in *Spisula solida*, *Spisula subtruncata* and *Mactra stultorum*.
