# Supplementary material for: Molecular Cytogenetics in Trough Shells (Mactridae, Bivalvia): Divergent GC-Rich Heterochromatin Content
Source: Genes (Basel). 2016 Aug 16;7(8):47. doi: 10.3390/genes7080047 (PMC4999835; doi:10.3390/genes7080047)
Supplement: Supplementary file 1 [file genes-07-00047-s001.docx]

Supplementary Materials: Molecular Cytogenetics in Trough Shells (Mactridae, Bivalvia): Divergent GC-Rich Heterochromatin Content

Daniel García-Souto, Concepción Pérez-García, Jack Kendall and Juan J. Pasantes


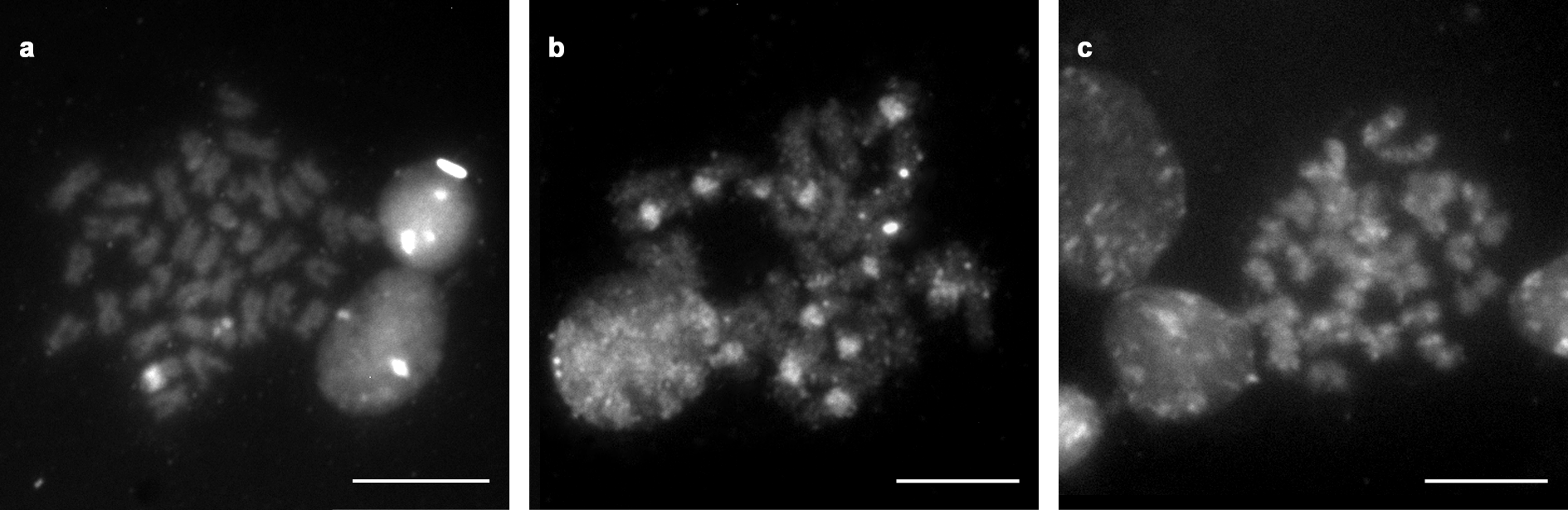


**Figure S1.** C-banding in *Spisula solida* (**a**); *Spisula subtruncata* (**b**); and *Mactra stultorum* (**c**).
